# Radiosynthesis and preliminary biological evaluation of *N*-(2-[^18^F]fluoropropionyl)-L-glutamine as a PET tracer for tumor imaging

**DOI:** 10.18632/oncotarget.9115

**Published:** 2016-05-03

**Authors:** Caihua Tang, Ganghua Tang, Siyuan Gao, Shaoyu Liu, Fuhua Wen, Baoguo Yao, Dahong Nie

**Affiliations:** ^1^ PET-CT Center, Department of Nuclear Medicine, The First Affiliated Hospital, Sun Yat-Sen University, Guangzhou, P.R. China; ^2^ Department of Nuclear Medicine, The Fifth Affiliated Hospital, Sun Yat-Sen University, Zhuhai, P.R. China

**Keywords:** [18F]FPGLN, glutamine, positron emission tomography, biologic evaluation, tumor imaging

## Abstract

In this study, radiosynthesis and biological evaluation of a new [^18^F]labeled glutamine analogue, *N*-(2-[^18^F]fluoropropionyl)-L-glutamine ([^18^F]FPGLN) for tumor PET imaging are performed. [^18^F]FPGLN was synthesized via a two-step reaction sequence from 4-nitrophenyl-2-[^18^F]fluoropropionate ([^18^F]NFP) with a decay-corrected yield of 30 ± 5% (*n*=10) and a specific activity of 48 ± 10 GBq/μmol after 125 ± 5 min of radiosynthesis. The biodistribution of [^18^F]FPGLN was determined in normal Kunming mice and high uptake of [^18^F]FPGLN was observed within the kidneys and quickly excreted through the urinary bladder. *In vitro* cell experiments showed that [^18^F]FPGLN was primarily transported by Na^+^-dependent system X_AG_^−^ and was not incorporated into proteins. [^18^F]FPGLN displayed better stability *in vitro* than that *in vivo*. PET/CT studies revealed that intense accumulation of [^18^F]FPGLN were shown in human SPC-A-1 lung adenocarcinoma and PC-3 prostate cancer xenografts. The results support that [^18^F]FPGLN seems to be a possible PET tracer for tumor imaging.

## INTRODUCTION

Although [^18^F]FDG is unsurpassed for the imaging of most tumors and is the current gold standard for cancer imaging by PET, a noticeable portion of these tumors are [^18^F]FDG PET-negative and go undetected [1]. Because of the low tumor aggressiveness and growth rate, relatively indolent tumors, such as bronchioloalveolar carcinoma, well-differentiated lung adenocarcinoma and prostate cancer, are known to have generally poor ^18^F-FDG uptake [2, 3]. It has been reported that [^18^F]FDG negative tumors may use a different metabolic pathway called glutaminolysis [4]. When the oncogene c-Myc is activated, the tumor cells may turn to glutamine as its major energy source, using the TCA cycle within the mitochondria to produce energy and cell building blocks [5, 6]. In addition, the tumor cells may likely use both glucolysis and glutaminolysis to generate energy for growth and survival. Several [^18^F]labeled glutamic acid and [^18^F]labeled glutamine analogues have been used for metabolic PET imaging of tumor in humans [7, 8]. Recently, 4 isomers of [^18^F]labeled 4-fluoroglutamine have been reported, and [^18^F](2S,4R)4-fluoroglutamine, one of the isomers, have demonstrated high tumor cell uptake and retention, but it is unstable and become defluorinated *in vivo*, resulting in suboptimal images [9].

**Figure 1:**
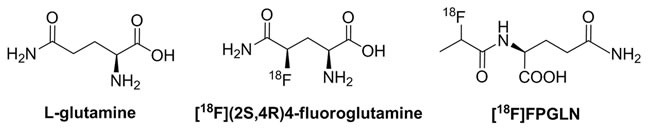
Chemical structures of L-glutamine, [^**18**^F](2S,4R)4-fluoroglutamine and N-(2-[^**18**^F]fluoropropionyl)-L-glutamine ([^18^F]FPGLN)

Our previous studies proposed the hypothesis that radiolabeled *N*-position amino acid analogues could function as potential PET tracers, which was confirmed by our early studies of *N*-(2-[^18^F]fluoropropionyl)-L-methionine ([^18^F]FPMET), which is a typical tracer for tumor imaging [10]. Although avid [^18^F]FPMET uptake was seen on S180 fibrosarcoma, A549 lung adenocarcinoma and PC-3 prostate cancer xenografts, its potential use in a clinical study was hampered by poor stability and a high background signal *in vivo*. Recently, a *N*-[^18^F]labeled L-glutamic acid analogue, *N*-(2-[^18^F]fluoropropionyl)-L-glutamate ([^18^F]FPGLU) was successfully synthesized as a potential novel PET tracer for glutamate metabolic imaging by our research group, which showed good tumor-to-background contrast in S180 fibrosarcoma, SPC-A-1 and LTEP-a-2 human lung adenocarcinoma mice models [11]. The objective of the present work was to radiosynthesize *N*-(2-[^18^F]fluoropropionyl)-L-glutamine ([^18^F]FPGLN), as shown in Figure 1, and to evaluate the potential value of this new PET imaging agent *in vitro* as well as in tumor-bearing nude mice models.

## RESULTS

### Radiosynthesis of [^18^F]FPGLN

**Figure 2:**
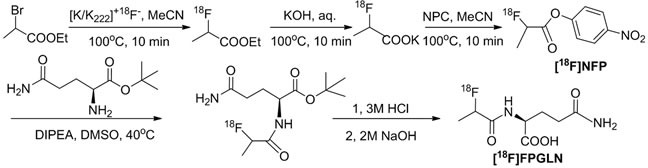
The scheme of radiosynthesis of [^**18**^F]FPGLN

**Figure 3:**
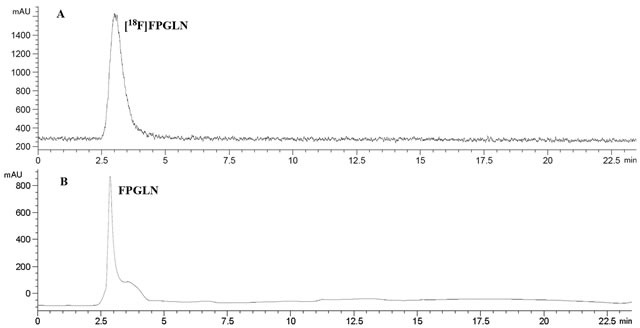
HPLC chromatograms of [^**18**^F]FPGLN **A.** and cold FPGLN **B.**

The scheme of radiosynthesis of [^18^F]FPGLN is shown in Figure 2. The radiochemical yield of [^18^F]FPGLN from [^18^F]NFP was 65 ± 10% (*n* = 10) for 30 min. The total decay-corrected radiochemical yield of [^18^F]FPGLN was 30 ± 5% (*n* = 10) from [^18^F]fluoride for 125 ± 5 min, with a specific activity of 48 ± 10 GBq/μmol. The identities of [^18^F]FPGLN were confirmed by comparison with non-radioactive reference compound FPGLN. The radiochemical purity of [^18^F]FPGLN was over 98%, and the retention time was approximately 2.5-4.0 min for [^18^F]FPGLN, as determined by radio-HPLC (Figure 3).

### Biodistribution study

The biodistribution data of [^18^F]FPGLN in normal mice is summarized in Table 1. Rapid and high uptake of radiotracer was observed within the kidneys but was quickly excreted through the urinary bladder, with 34.95% and 1.49% of the injected dose per gram of tissue (% ID/g) in the kidneys at 5 min and 120 min, respectively. The uptake of [^18^F]FPGLN in blood was relative high and decreased gradually within the observed time from 4.02 to 2.13% ID/g at 5 and 120 min, respectively. A moderate uptake of radioactivity was shown in the liver and lung, with 3.38% and 3.11% ID/g at 5 min, which decreased slowly to 1.51% and 1.38% ID/g after 120 min, respectively. There were relatively low uptake levels of [^18^F]FPGLN in other organs of interest, for example, the heart, pancreas, intestine, stomach, spleen, bone and muscle. Moreover, the brain was the organ with the lowest uptake level in our study. The bone uptake of [^18^F]FPGLN at 120 min was not significantly higher than that at 60 min (*P* > 0.05), therefore, there is no defluorination of [^18^F]FPGLN observed *in vivo*.

**Table 1:** Biodistribution (% ID/g) of [^18^F]FPGLN in normal mice

	5 min	30 min	60 min	120 min
**Blood**	4.02 ± 0.10	2.80 ± 0.15	2.27 ± 0.27	2.13 ± 0.21
**Brain**	1.19 ± 0.11	1.69 ± 0.10	1.42 ± 0.26	1.27 ± 0.15
**Heart**	2.39 ± 0.15	2.64 ± 0.83	2.15 ± 0.59	1.62 ± 0.26
**Lung**	3.11 ± 0.74	2.27 ± 0.59	1.63 ± 0.46	1.38 ± 0.26
**Liver**	3.38 ± 0.42	2.67 ± 0.37	1.80 ± 0.43	1.51 ± 0.22
**Spleen**	1.99 ± 0.08	1.85 ± 0.19	1.50 ± 0.36	1.40 ± 0.13
**Kidney**	34.95 ± 1.19	11.23 ± 1.69	2.28 ± 0.86	1.49 ± 0.28
**Pancreas**	2.14 ± 0.52	1.45 ± 0.12	1.14 ± 0.50	0.97 ± 0.21
**Stomach**	1.71 ± 0.54	1.35 ± 0.04	1.24 ± 0.38	1.10 ± 0.15
**Intestine**	2.14 ± 0.23	2.28 ± 0.38	1.57 ± 0.39	1.44 ± 0.20
**Muscle**	2.12 ± 0.72	1.32 ± 0.40	1.58 ± 0.28	1.46 ± 0.25
**Bone**	2.19 ± 0.25	1.86 ± 0.47	1.80 ± 0.03	2.13 ± 0.21

Note: Data are average % ID/g ± SD (*n* = 4).

### Transport characterization studies

**Figure 4:**
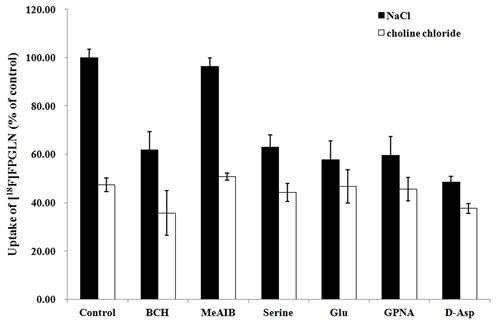
Uptake of [^**18**^F]FPGLN in SPC-A-1 cells in presence of inhibitors in the medium of presence and absence of Na^+^ Values are given as percentage (mean ± SD, *n* = 15) of uptake in cells that were incubated with or without inhibitors including BCH, MeAIB, serine, L-glutamate(Glu), L-γ-glutamyl-p-nitroanilide (GPNA) and D-aspirate ( D-Asp) in Na^+^-containing (NaCl) or choline chloride buffer.

The result of the competitive inhibition experiment in the presence or absence of Na^+^ is shown in Figure 4. In the presence of Na^+^, system X_AG_^−^ inhibitor D-aspirate and L-glutamate (an inhibitor for system X_C_^−^ or X_AG_^−^), inhibited uptake of [^18^F]FPGLN by 52% and 42%, respectively (*P* < 0.01, *n* = 15), indicated that Na^+^-dependent X_AG_^−^ possibly closely correlated with the transporter of [^18^F]FPGLN. The uptake of [^18^F]FPGLN was suppressed by L-serine, inhibitor for system ASC, by 37% (*P* < 0.05, *n* = 15). While, L-γ-glutamyl-p-nitroanilide (GPNA), specific inhibitor for system ASCT2, approximately reduced the uptake of [^18^F]FPGLN by 40% (*P* < 0.05, *n* = 15), suggesting Na^+^-dependent system ASC (especially ASCT2) may also involved in the transporter of [^18^F]FPGLN. The addition of 2-amino-2-norbornanecarboxylic acid (BCH), decreased the uptake of [^18^F]FPGLN by 38%, suggesting that [^18^F]FPGLN transport was partly mediated through Na^+^-dependent system B^0+^ (*P* < 0.05, *n* = 15). System A inhibitor MeAIB did not markedly suppress the uptake of [^18^F]FPGLN (*P* > 0.05, *n* = 15). Compared with that in the presence of Na^+^ control group, the uptake of [^18^F]FPGLN in the absence of Na^+^ control group was decreased to 47.4% (*P* < 0.01, *n* = 15). BCH reduced the uptake of [^18^F]FPGLU by 25% (*P* < 0.05, *n* = 15), however other inhibitors (MeAlB, serine, Glu, GPNA and D-Asp) did not markedly inhibit the uptake of [^18^F]FPGLN, indicating that Na^+^-independent system L also involved in the uptake of [^18^F]FPGLN. Therefore, [^18^F]FPGLN was primarily transported by Na^+^-dependent system X_AG_^−^, Na^+^-dependent ASC (especially ASCT2) and system B^0+^ were partly involved in the uptake of [^18^F]FPGLN in SPC-A-1 cells, with minor involvement of Na^+^-independent system L and almost no involvement of systems A transporter.

### Protein incorporation

SPC-A-1 cells were incubated with [^18^F]FPGLN for 30 min, then trichloroacetic acid was used to precipitate them, there was 1.3% of the radioactivity in the acid precipitable fraction. The result of protein incorporation demonstrated that there was almost no incorporation of [^18^F]FPGLN into the protein, which was similar to that of many ^18^F-labeled non-protein-composition amino acids tracers (e.g. [^18^F]FPMET, [^18^F]FPGLU and 4-[^18^F]fluoroglutamic acid) [10–12] and was different from that of [^18^F](2S,4R)4-fluoroglutamine, which has a high incorporated protein ratio. The protein incorporation of [^18^F](2S,4R)4-fluoroglutamine in 9L and SF188 cells was 29% and 12% at 30 min, and 62% and 72% at 120 min, respectively [13]. The results strongly suggested that uptake of [^18^F]FPGLN in tumor could predominantly reflect the amino acid transport rather than protein incorporation.

### Stability of [^18^F]FPGLN *in vitro* and *in vivo*

**Figure 5:**
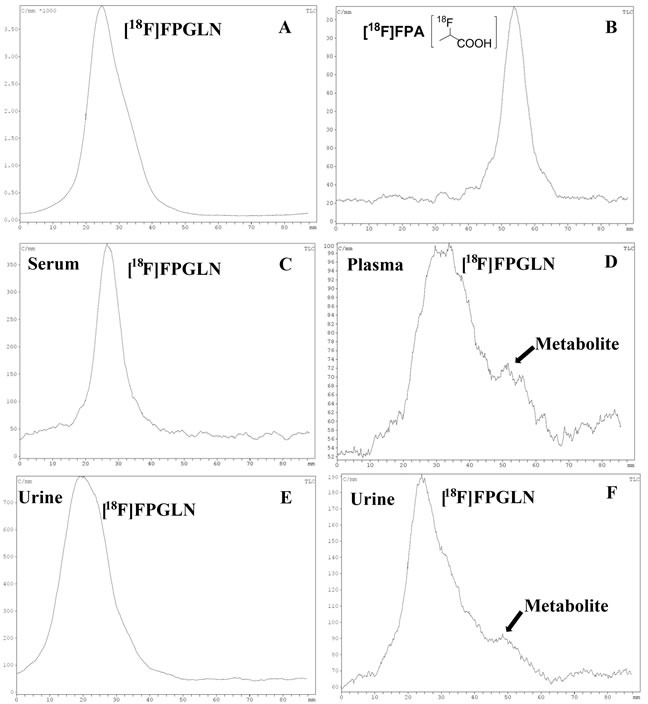
Analysis the stability of [^**18**^F]FPGLN by reversed-phase thin-layer chromatography (TLC) *in vitro* and *in vivo* **A.** [^18^F]FPGLN injected solution. **B.** [^18^F]FPA injected solution. **C.** [^18^F]FPGLN co-cultured with mouse serum for 2 h. **D.** Plasma collected at 0.5 h post-injection of [^18^F]FPGLN. E and F. Urine collected at 0.5 h **E.** and 1 h **F.** post-injection of [^18^F]FPGLN.

The results of radio thin-layer chromatography (radio-TLC) of the samples are shown in Figure 5. For the radioactivity level of the plasma collected after 1 h was too low, the radio-TLC was only used to analyze the metabolites for the plasma up to 30 min. We found that [^18^F]FPGLN was stable *in vitro* because more than 95% of the [^18^F]FPGLN remained intact after co-cultured with mouse serum for 2 h (Figure 5C). *In vivo* metabolism of [^18^F]FPGLN, because the “dot” position of the samples and the distant of the solvents developed were not exactly the same, the peak positions of [^18^F]FPGLN were not fully consistent in each thin-layer chromatography. However, the Rf values of [^18^F]FPGLN were basically consistent. More than 70% of the [^18^F]FPGLN remained intact in the plasma at 0.5 h post-injection(Figure 5D), and main parent [^18^F]FPGLN was detected in the urine at 0.5 h and 1 h after [^18^F]FPGLN administration (Figure 5E and 5F). However, some unknown metabolites can also be detected in the plasma at 0.5 h post-injection (Figure 5D) and in the urine at 1 h post-injection(Figure 5F). The results indicated that [^18^F]FPGLN was not enough stable *in vivo*.

### PET studies

**Figure 6:**
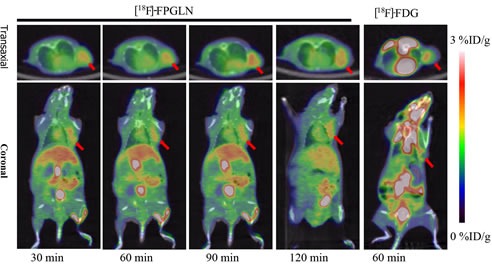
Decay-corrected whole-body PET/CT fusion images acquired at different time points PET/CT fusion images of SPC-A-1 lung adenocarcinoma-bearing mouse static scans at 30, 60, 90 and 120 min after the injection of [^18^F]FPGLN. The same tumor-bearing mouse static scan at 60 min after injection of [^18^F]FDG was also shown (The red arrows indicate the tumors).

Small-animal PET/CT imaging using [^18^F]FPGLN or [^18^F]FDG was performed on SPC-A-1 lung adenocarcinoma and PC-3 prostate cancer xenografts mice models (*n* = 3 per group). The PET/CT fusion images of SPC-A-1-bearing models are shown in Figure 6. High uptake of [^18^F]FPGLN in SPC-A-1 lung adenocarcinoma xenograft was observed at 30 min post-injection. The tumors were clearly visible with high contrast to the background (muscles and lungs) during the 2 h imaging protocol. Regions of interest (ROIs) from the whole organ on the coronal images were measured so that the accumulation of the radioactivity in the small-animal PET scans could be quantified. The tumor to muscle (T/M) and tumor to lung (T/L) relative uptake ratios of [^18^F]FPGLN reached the peak (2.29 ± 0.05, 3.80 ± 0.23) at 90 min post-injection. The result demonstrated that [^18^F]FPGLN may be an useful PET tracer for lung adenocarcinoma imaging.

**Figure 7:**
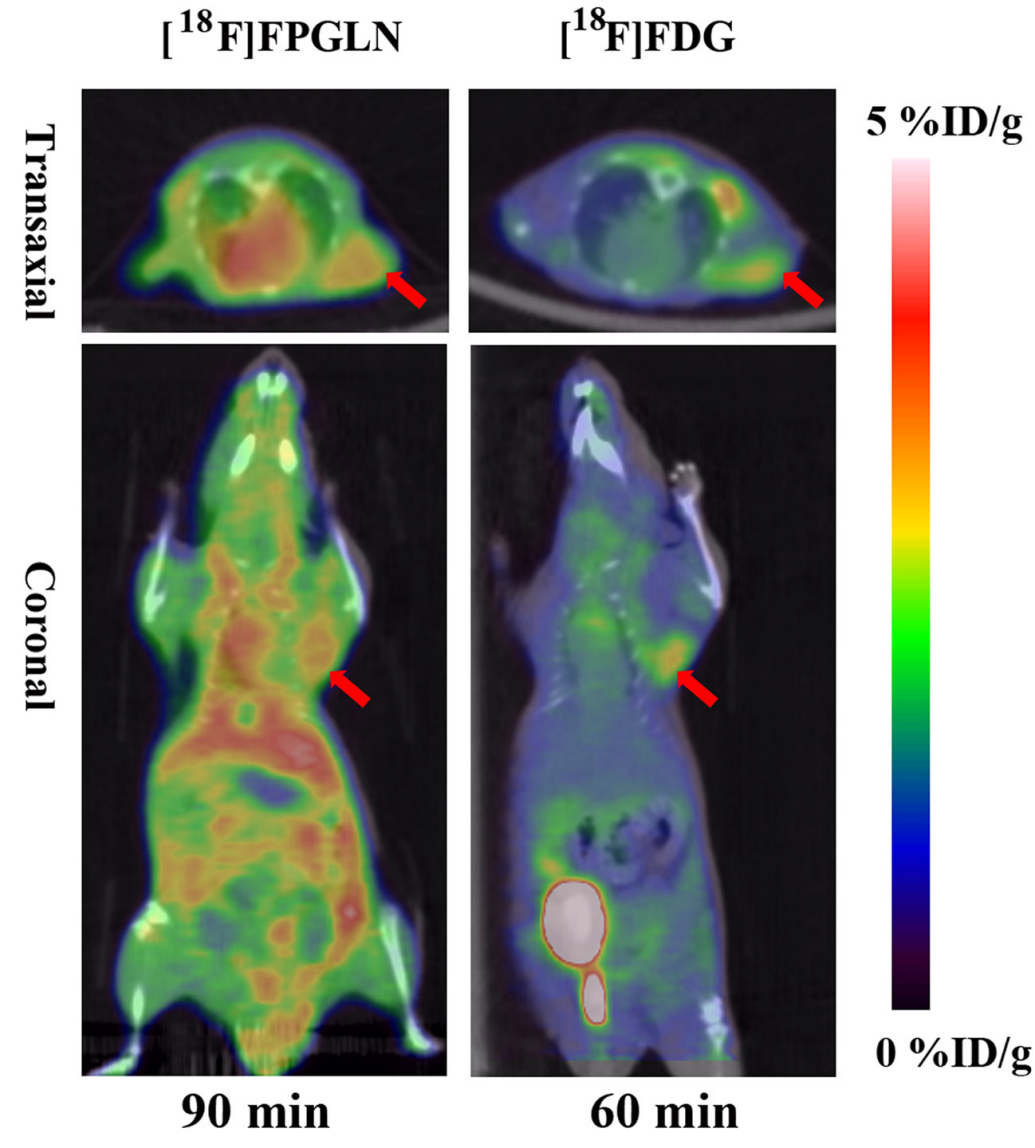
Decay-corrected whole-body PET/CT fusion images of PC-3 prostate cancer-bearing mice after the injection of [^**18**^F]FPGLN or [^**18**^F]-FDG The red arrows indicate the tumors.

PET/CT fusion images with [^18^F]FPGLN or [^18^F]FDG of PC-3 prostate cancer-bearing mice models are shown in Figure 7. Although intense uptake of both [^18^F]FPGLN and [^18^F]FDG were seen in the PC-3 prostate cancer xenografts. The uptake between [^18^F]FPGLN and [^18^F]FDG in PC-3 xenografts has no significant difference at 1 h post-injection (2.95 ± 0.26 %ID/g *vs*. 2.80 ± 0.14 %ID/g, *n* = 3, P > 0.05). However, because of the relatively high uptake of [^18^F]FPGLN in muscles, the tumor to muscle uptake ratio was not so high (T/M = 1.88 ± 0.23) in PC-3 models.

## DISCUSSION

Glutamine plays important functions in tumor cells (such as protecting against oxidative stress, mediating new signal transduction pathways, enhancing the tolerance of changes in the microenvironment) and promotes tumor growth [14,15]. In this study, we synthesized a novel N-[^18^F]labeled L-glutamine analogue, [^18^F]FPGLN, for tumor imaging with PET.

The biodistribution of [^18^F]FPGLN in normal mice was similar to [^18^F]labeled glutamic acid analogue [^18^F]FPGLU [11], both with radioactivity rapidly accumulated in kidneys and quickly excreted through the urinary-bladder route. A moderate uptake of radioactivity was shown in the liver, which is an important site of glutamine metabolism. The radioactivity in other tissues was relatively low during the entire observation time, suggesting that [^18^F]FPGLN had a lower background signal *in vivo*. The uptake of bone was very low during the 2 hours protocol, with 2.19 and 2.13% ID/g at 5 and 120 min, respectively. Thus, there is no defluorination of [^18^F]FPGLN *in vivo*. It is different from that of [^18^F](2S,4R)4-fluoroglutamine, with bone (femur) uptake gradually rising from 3.93% ID/g at 2 min to 19.40 % ID/g at 240 min after injection [13].

The uptake experiments showed that the transport characteristics of [^18^F]FPGLN in SPC-A-1 cells was partly similar to that of [^18^F]FPGLU, which was mediated through system X_AG_^−^, ASC, and Na-independent system L, with X_AG_^−^ playing a dominant role [11]. The transport of [^18^F]FPGLN was also partly similar to that of [^18^F](2S,4R)4-fluoroglutamine, whose transport involved systems L, ASC, and N [13]. Tumor cells upregulate one or more of the amino acid transporters to satisfy their demand for amino acids. Several amino acid transporters, such as LAT1( system L), ASCT2(system ASC), ATB^0,+^(system B^0,+^) have been found overexpressed in a variety cancers [16–18]. In addition, stably expression of glutamate transporters (System X_AG_^−^) has been found on human brain tumors [19] and prostate cancer cells [20]. Thus, the high uptake of [^18^F]FPGLN in SPC-A-1 lung adenocarcinoma and PC-3 prostate cancer xenografts may closely related to the upregulation of amino acid transporters mentioned above.

Although the [^18^F]FPGLN was stable *in vitro*, it was not enough stable *in vivo*. Some metabolites of [^18^F]FPGLN can be detected in the plasma at 0.5 h and urine at 1 h post-injection. The results showed that the Rf value of the metabolites of [^18^F]FPGLN in the plasma and urine was partly similar to that of [^18^F]FPA (Figure 5A and 5B). Therefore, we deduce that the amide bond of [^18^F]FPGLN may be cleaved by some enzymes in animals after 0.5 h post-injection. [^18^F]FPA may be a metabolic product of [^18^F]FPGLN, but which needs to be further confirmed. As [^18^F]FPA is also highly uptaken in tumor [21], we suggest that uptake mechanism of [^18^F]FPGLN may involve in amino acid transport at the early stage of PET imaging and [^18^F]FPA metabolism at the late stage of tumor PET imaging. However, further research will be performed to confirm this hypothesis in the near future.

PET images of SPC-A-1 tumor bearing models showed radioactivity selectively accumulated in tumors with high tumor to background uptake ratios. Although radioactivity also selectively accumulated in PC-3 xenografts, diffuse uptake of [^18^F]FPGLN was shown in Figure 7 and the tumor to muscle uptake ratios of [^18^F]FPGLN were not better than that of [^18^F]FDG. The possible reason is that the uptake of [^18^F]FPGLN in PC-3 tumor was not so high, while the uptake of muscle was relatively high, and [^18^F]FPGLN may have been partly metabolized at 90 min post-injection. Due to low accumulation of [^18^F]FPGLN in normal brain tissues, the uptake ratios of tumor to brain tissue of [^18^F]FPGLN were significantly higher than that of [^18^F]FDG at 60 min post-injection (2.09 ± 1.10 *vs*. 0.63 ± 0.16, *n* = 3, *P* < 0.01), further research will be performed to evaluate this new PET tracer for brain tumor (such as glioma) imaging.

## CONCLUSIONS

In this study, we have synthesized a novel N-position [^18^F]radiolabeled L-glutamine analogue, [^18^F]FPGLN, as a potential PET tracer for tumor imaging. Rapid and high uptake of [^18^F]FPGLN is observed within the kidneys and quickly excreted through the urinary bladder. [^18^F]FPGLN is primarily transported through Na^+^-dependent system X_AG_^−^ in SPC-A-1 cells and is not incorporated into proteins. [^18^F]FPGLN demonstrates better stable *in vitro* than *in vivo*. Intense accumulation of [^18^F]FPGLN is observed in the PET imaging of SPC-A-1 lung adenocarcinoma and PC-3 prostate cancer-bearing mice. The results suggest that [^18^F]FPGLN seems to be a possible metabolic PET tracer for tumor imaging.

## MATERIALS AND METHODS

### General information

All reagents, unless otherwise specified, were of analytical grade and commercially available. All chemicals obtained commercially were used without further purification. Sep-Pak light QMA and Plus C18 cartridges were obtained from Waters Corporation (Milford, MA, USA). Dimethylsulfoxide (DMSO), acetonitrile (MeCN), *N, N*-diisopropylethylamine (DIPEA), bis(4-nitrophenyl)carbonate (NPC), trifluoroacetic acid (TFA), Kryptofix 2.2.2, L-glutamine t-butyl ester hydrochloride were purchased from Sigma-Aldrich (Milwaukee, WI, USA). [^18^F]FDG was prepared as previously reported [22]. Non-radioactive reference compounds [^19^F]fluoropropionyl-L-glutamine (FPGLN) was offered by professor Jiang Shende in School of Pharmaceutical Science and Technology, Tianjin University. FPGLN was characterized by electrospray mass spectrometry, ^1^H-NMR spectroscopy and ^19^F spectroscopy. ^1^H NMR (MeOD-d4, δ):7.14 (m, 1H), 5.02 (m, 1H), 4.35 (m, 1H), 2.16 (dt, 2H, J = 30.7, 7.2 Hz), 2.14 (m, 1H), 1.92 (m, 1H), 1.39-1.47 (m, 3H). ^19^F NMR (MeOD-*d*4, δ): −184.36 - −184.63 (m). MS (ESI) m/z (%)219.9[M-H]^−^.

Before used for radiosynthesis, the Sep-Pak light QMA cartridges were preconditioned with 10 mL 8.4% NaHCO_3_ aqueous and 10 mL water, and the Sep-Pak plus C18 cartridges were preconditioned with 10 mL ethanol and water, respectively. HPLC separation was carried out at the PET-MF-2V-IT-I synthesizer module (PET Co. Ltd., Beijing, China) built-in HPLC system with a semipreparative reverse-phase C18 column (10×250 mm) equipped with a UV detector (Alltech 201, USA) and a radioactivity detector (PET Co. Ltd., China). The mobile phase was 50% solvent A (0.1% TFA in water): 50% solvent B (0.1% TFA in MeCN) and the flow rate was 4 mL/min. Analytical HPLC and radio thin layer chromatography (radio-TLC) were used for checking of radiochemical purities and radiochemical stability of [^18^F]FPGLN. An Agilent 1200 Series HPLC system equipped with a ZORBAX Eclipse XDB-C18 analytic column (4.6×150 mm, Agilent) was used for analytical HPLC, and the flow rate was 1 mL/min. The gradient program was performed by a similar method as previously described [10]. Radioactivity was measured by a calibrated ion chamber (Capintec CRC-15R) or a gamma counter (γ-counter) (GC-1200, USTC Chuangxin Co. Ltd. Zonkia Branch, China). The elution profile was detected with an ultraviolet detector (Agilent interface 35900E, Agilent Technologies, USA) at 210 nm and a B-FC-3200 high energy PMT Detector (Bioscan. Inc, Washington DC, USA). Radio-TLC was developed on silica gel plate by the mixed solvent of MeCN/H_2_O (v/v = 9/1). No-carrier-added [^18^F]fluoride was obtained through the nuclear reaction ^18^O(p, n)^18^F by irradiation of more than 95% [^18^O]enriched water target with 10 MeV proton beam on the Cyclone 10/5 cyclotron (IBA Technologies, Belgium). Inveon small-animal PET/computed tomography (CT) scanner (Siemens) was used to perform the small-animal PET/CT imaging of tumor-bearing mice.

### Radiosynthesis of [^18^F]FPGLN

The radiosynthesis of [^18^F]FPGLN *via* a two-step reaction sequence from 4-nitrophenyl-2-[^18^F]fluoropropionate ([^18^F]NFP) was similar to that of *N*-(2-[^18^F]-fluoropropionyl)-L-glutamate [^18^F]FPGLU described recently [11]. The fully automated synthesis of 4-nitrophenyl-2-[^18^F]fluoropropionate ([^18^F]NFP) was carried out on the PET-MF-2V-IT-I synthesizer *via* a simplified three step, one-pot procedure has been described in detail by the earlier paper [10]. After dried with a sodiumsulfate (Na_2_SO4) cartridge, anhydrous [^18^F]NFP was added to a solution of L-glutanine t-butyl ester hydrochloride ( 200-300 μg) in DMSO (200 μL) and DIPEA (20 μL). The reaction mixture was heated at 40°C for 10 min, then the reaction was quenched by adding of 5% acetic acid (800 μL), and diluted with 2 mL water. The *N*-(2-[^18^F]fluoropropionyl)-L-glutamine t-butyl ester was trapped on a C18 cartridge and washed with 8 mL water. Then, *N*-(2-[^18^F]fluoropropionyl)-L-glutamine t-butyl ester was eluted by ether (5 mL) to a new tube, and the ether was evaporated under nitrogen flow at room temperature. HCl (3 M, 100 μL) solution was added to the residue, and *N*-(2-[^18^F]fluoropropionyl)-L-glutamine t-butyl ester was hydrolyzed at 110°C for 10 min, then the solution was cooled to room temperature and neutralized with 2 M NaOH (150 μL) solution. Finally, the [^18^F]FPGLN product was collected in a vented sterile vial through a 0.22 μm Millipore filter for further studies.

### Cells culture and animal models

The SPC-A-1 human lung adenocarcinoma cells and PC-3 prostate cancer cells lines were obtained from Shanghai Institute of Cellular Biology of Chinese Academy of Sciences (Shanghai, China). The cells were cultured in culture flasks containing RPMI 1640 medium supplemented with 10% fetal bovine serum and 1% penicillin/streptomycin at 37°C in a humidified atmosphere of 5% CO_2_ and 95% air. The SPC-A-1 cells in the logarithmic growth phase were used for the *in vitro* experiments of the transport assay and protein incorporation. 24 hours prior to the experiments, the SPC-A-1 cells were trypsinized and were seeded into 24-well plates (2.0 × 10^5^ cells/well).

All animal experimental studies were approved by the Institutional Animal Care and Utilization Committee (IACUU) of the First Affiliated Hospital, Sun Yat-Sen University (approval No.[2013]A-173). All efforts were made to minimize animal suffering, to reduce the number of animals used, and to use alternatives to *in vivo* techniques, if available. Normal Kunming mice (female, 4-6 weeks old, 20-25g) for biodistrubution studies of [^18^F]FPGLN and BALB/c nude mice (4-6 weeks old, 18-22g) for tumor-bearing models were obtained from Laboratory Animal Center of Sun Yat-Sen University (Guangzhou, China). The SPC-A-1 and PC-3 tumor-bearing models were made using the previously described methods [11, 23]. Tumor cells (1-2×10^7^) were injected subcutaneously on left shoulder flank and allowed to grow for 2 to 3 weeks. Micro PET/CT scans were done when the tumor diameter reached 6-10 mm. The mice were housed 5 animals per cage under standard laboratory conditions at 25°C and 50% humidity and allowed free access to food and water.

### Biodistribution study

To reduce the costs and numbers of animal models used, normal Kunming mice was used to replace tumor-bearing mice models in the biodistribution study. The Kunming mice were divided into four groups (*n* = 4 per group), each mouse was injected with 0.74-1.48 MBq (20-40μCi) of [^18^F]FPGLN in 100-200 μL of phosphate-buffered saline (PBS) through the tail vein. The animals were anesthetized with 5% chloral hydrate solution (6 mL/kg) before injection of radiotracer and remained anesthetized throughout the study. The animals were sacrificed by cervical dislocation at various times (5, 30, 60 and 120 min) postinjection of the radiotracer. Blood was obtained through the eyeballs, tissue samples of interest (brain, heart, lung, liver, spleen, kidney, pancreas, stomach, small intestine, muscle and right thigh bone) were rapidly dissected and weighed, and radioactivity was counted with a gamma (γ) counter (GC-1200, USTC Chuangxin Co. Ltd. Zonkia Branch, China). All measurements were background-subtracted and decay-corrected to the time of injection and then averaged. Data were expressed as a percentage of the injected dose per gram of tissue (% ID/g).

### *In vitro* transport mechanism studies

To investigate the transport mechanisms involved in the uptake of [^18^F]FPGLN, we conducted a series of competitive inhibition studies using SPC-A-1 cells line. Various types of inhibitors: 2-amino-2-norbornanecarboxylic acid (BCH) for system L, *N*-methyl-2-amino-isobutyric acid (MeAIB) for system A, L-glutamate for system X_AG_^−^ and X_C_^−^, D-aspartate for system X_AG_^−^, L-serine for system ASC, L-γ-glutamyl-p-nitroanilide (GPNA) specially for transporter ASCT2 were used for the competitive inhibition studies. All experiments were performed in the presence and absence of Na^+^, and sodium salts were replaced by choline chloride in Na^+^-free experiments. The medium was aspirated and the cells were washed 3 times with 1 mL warm PBS (containing 0.90 mM of Ca^2+^ and 1.05 mM of Mg^2+^). The inhibitors dissolved in PBS solution and were added to each well (0.2 mL/well) with the final concentration of 15 mmol/L. [^18^F]FPGLN was also dissolved in PBS solution and was added to each well (74-111 KBq/0.2 mL/well). After incubated with [^18^F]FPGLN at 37°C for 10 min, the radioactive medium was removed and the cells were washed 3 times with ice-cold PBS without Ca^2+^ and Mg^2+^. Then, the cells were dissolved in 0.5 mL of 1 N NaOH and the activity was measured by γ counter (GC-1200, USTC Chuangxin Co. Ltd. Zonkia Branch, China). The cell lysate (100 μL) was used for determination of protein concentration by modified Lowry protein assay. The uptake data are based on the amount of activity added to each well and the total amount of protein in each well. Finally, the data are normalized in reference to the wells without any inhibitor in PBS solution (Na^+^). Each experiment was done in triplicate, averaged and was repeated 5 times on different days.

### Protein incorporation

The method of determining the extent of protein incorporation of [^18^F]FPGLN was performed according to the similar method reported earlier [11]. For incorporation of [^18^F]FPGLN into proteins experiments, the SPC-A-1 cells were respectively incubated with 400 μL(185-296 KBq) [^18^F]FPGLN at 37°C for 30 min. At the end of incubation, the radioactive medium was removed. The cells were washed three times with ice cold PBS (1.0 mL, pH = 7.4) and detached with 0.5 mL ethylenediaminetetraacetic acid (1%). Then, the samples were transferred into new tubes and 0.5 mL 20% trichloroacetic acid (TCA) was added. 10 min later, the samples were centrifuged at 13000 rpm for 5 min. The supernatant was removed, and the pellet was washed three times with ice-cold PBS. The radioactivity in both the supernatant and the pellet were measured by a γ counter. Protein incorporation was calculated as the percentage of acid precipitable radioactivity.

### Stability *in vitro* and *in vivo*

Because the amide bond of [^18^F]FPGLN can be cleaved *in vivo* by some enzymes to produce 2-[^18^F]fluoropropionate ([^18^F]FPA). It is necessary to identify the [^18^F]FPGLN and the possible metabolic product [^18^F]FPA. The retention time of [^18^F]FPA was a little similar to that of [^18^F]FPGLN with radio-HPLC, but their Rf values were different using radio-TLC analysis (Figure 5A and 5B). Thus, only radio-TLC was used to analysis the stability of [^18^F]FPGLN *in vitro* and *in vivo*. [^18^F]FPA was produced according to our pervious methods [24] and its chemical structure was shown in Figure 5B. *In vitro* stability experiment, a sample of [^18^F]FPGLN (0.74 MBq, 20 μL) dissolved in normal saline was added to 200 μL of mouse serum and incubated at 37°C for 30, 60 and 120 min. An aliquot of the serum sample was analyzed by radio-TLC to analyze the stability of [^18^F]FPGLN in mouse serum within 2 h. *In vivo* stability experiment, Kunming mice were anesthetized with 5% chloral hydrate solution (6 mL/kg) and were injected with a dose of approximately 18.5 MBq (0.5 mCi) of [^18^F]FPGLN in 200 μL of normal saline *via* the tail vein. The mice were sacrificed at 0.5 h and 1 h after injection. Blood was obtained from the eyeballs, centrifuging (13000 rpm, 5 min). Both the plasma and urine were used to analyze the metabolic fate of [^18^F]FPGLN *in vivo* by radio-TLC chromatograms.

### PET study and imaging analysis

PET/CT imaging studies were performed on SPC-A-1 and PC-3 tumor-bearing nude mice using the Inveon small-animal PET/CT scanner (Siemens). 3.70-7.40 MBq (100-200 μCi) [^18^F]FPGLN in 200 μL of PBS was injected intravenously in conscious animals *via* the tail vein. The mice were anesthetized with 5% chloral hydrate solution (6 mL/kg) ten minutes later and were placed on a heating pad to maintain body temperature throughout the procedure. Then, imaging acquisition started with a low-dose CT scan and followed by a 10 min per bed position PET scan immediately. The CT scan was used for attenuation correction and localization of the lesion site. PET image acquisition was performed at 30, 60, 90, 120 min after intravenous injection of [^18^F]FPGLN in 3-dimensional mode. For a comparative study, [^18^F]FDG PET/CT imaging scan was also performed on mice at 60 min after intravenous injection of [^18^F]FDG (3.7 MBq). The animals were kept fasting for at least 4 h and were anesthetized with 5% chloral hydrate solution (6 mL/kg) before injection of [^18^F]FDG and remained anesthetized throughout the study. The images were reconstructed and the regions of interest (ROIs) were drawn over the tumor and muscle of the right thigh on decay-corrected whole-body coronal images using Inevon Research Workplace 4.1 software. The quantification was performed according the methods described previously [11]. An imaging ROI-derived % ID/g was obtained and the tumor to background relative uptake ratio was calculated finally.

### Statistical analysis

SPSS software, version 16.0 (SPSS Inc.), for Windows (Microsoft) was used to perform the statistical analysis. All data were expressed as mean ± SD. Comparisons between conditions were performed using the unpaired, 2-tailed Student *t* test. A *P* value of less than 0.05 was considered to indicate statistical significance.
